# Inducing Favorable Cation Antisite by Doping Halogen in Ni‐Rich Layered Cathode with Ultrahigh Stability

**DOI:** 10.1002/advs.201801406

**Published:** 2018-12-12

**Authors:** Chunli Li, Wang Hay Kan, Huilin Xie, Ying Jiang, Zhikun Zhao, Chenyou Zhu, Yuanhua Xia, Jie Zhang, Kang Xu, Daobin Mu, Feng Wu

**Affiliations:** ^1^ School of Material Science & Engineering Beijing Key Laboratory of Environmental Science and Engineering Beijing Institute of Technology 100081 China; ^2^ Collaborative Innovation Center of Electric Vehicles in Beijing 100081 China; ^3^ Institute of High Energy Physics China Spallation Neutron Source Dongguan Guangdong 523000 China; ^4^ Key Laboratory of Neutron Physics Institute of Nuclear Physics and Chemistry China Academy of Engineering Physics Mianyang 621999 China; ^5^ Electrochemistry Branch Sensor and Electron Directorate U. S. Army Research Laboratory Adelphi MD 20783 USA

**Keywords:** antisite, halogen doping, neutral diffraction, nickle‐rich layered materials

## Abstract

The cation antisite is the most recognizable intrinsic defect type in nickel‐rich layered and olivine‐type cathode materials for lithium‐ion batteries, and important for electrochemical/thermal performance. While how to generate the favorable antisite has not been put forward, herein, by combining first‐principles calculation with neutron powder diffraction (NPD) study, a defect inducing the favorable antisite mechanism is proposed to improve cathode stability, that is, halogen substitution facilitates the neighboring Li and Ni atoms to exchange their sites, forming a more stable local octahedron of halide (LOSH). According to the mechanism, it is demonstrated by NPD that F‐doping not only induces the antisite formation in layered LiNi_0.85_Co_0.075_Mn_0.075_O_2_ (LNCM), but also increases the antisite concentration linearly. F substitution (1%) induces 5.7% antisite, and it displays an excellent capacity retention of 94% at 1 C for 200 cycles under 25 °C, outstanding high temperature cyclability (153.4 mAh·g^–1^ at 1 C for 120 cycles under 55 °C). The onset decomposition temperature increases by 48 °C. The ultrahigh cycling/thermal stability is attributed to the stronger LOSH, and it keeps the structural integrity after long cycling and develops an electrostatic repulsion force between oxygen layers to increase the lattice parameter *c*, which benefits Li‐ion migration.

Currently, LiNi_1‐_ *_x_*_‐_ *_y_*Co*_x_*Mn*_y_*O_2_ (LNCM) are the mainstream cathode materials used in Li‐ion batteries, whose diversified variations LiNi_1/3_Co_1/3_Mn_1/3_O_2_, LiNi_0.5_Co_0.3_Mn_0.2_O_2_, and LiNi_0.6_Co_0.2_Mn_0.2_O_2_ can deliver the high energy densities in the range of 150–220 wh·kg^−1^.^1^, ^2^, ^3^, ^4^ With the rising demand from the electric vehicles, higher capacity (≈200 mAh·g^−1^) Ni‐rich LiNi_0.8_Co_0.1_Mn_0.1_O_2_ (Ni‐rich LNCM) cathode has been proposed to be coupled with Li metal anode, promising an energy density of 400 wh·kg^−1^. However, Ni‐rich LNCM severely suffers from poor cycle life and thermal stability in comparison with LiFePO_4_ or LiMn_2_O_4_ cathodes due to structure instability. Extensive studies have been conducted to elucidate the structural stability of LNCM cathodes. By calculating the total energies of Li*_x_*CoO_2_, Li*_x_*NiO_2_, and Li*_x_*Mn_2_O_4_ under different delithiation stages,^5^ Ceder and co‐workers revealed that the decomposition of Li*_x_*NiO_2_ experiences a two‐steps reaction: the first of which is exothermic due to the transformation of lithium transition metal oxides from layered structure to a more stable spinel structure, and the second is an endothermic process induced by further transformation from spinel structure to rock‐salt structure, accompanied by oxygen releasing. Besides these structural phase transition during charging/discharging, defects have a great influence on the performance of layered LNCM materials; in general, there are three types of defects: cation antisite, extra Ni, and oxygen vacancy. Cation antisite is considered as the most recognizable intrinsic defect to impact on nickel‐rich layered and olivine‐type phosphates cathode materials (Li and Fe/Mn ions exchange positions in LiFe_0.5_Mn_0.5_PO_4_).^6^ Because of the similar ionic radii of Li^+^ (0.076 nm) and Ni^2+^ (0.069 nm) in LNCM, Ni^2+^ is more likely to diffuse into Li layer and occupy Li sites, which usually occurs in the synthesis process.^7^ Thus, many researchers pay attention to the formation of cation antisite defects.^8^, ^9^ A recent theoretical study demonstrated that the defect formation energies in LiNiO_2_ are very low and the presence of Ni in the Li layer can be ascribed to the 180° Ni–O–Ni super exchange interaction.^10^ It is not easy to detect the antisite defect by using conventional experimental methods (X‐ray powder diffraction [XRD], scanning transmission electron microscopy (STEM)). Xiao et al. uncovered the antisite in LiNi*_x_*Mn*_y_*Co*_z_*O_2_ via applying neutron powder diffraction (NPD) experiments and magnetization measurements.^11^

It is controversial whether the antisite is favorable for LNCM cathode material. Most studies think Ni at Li sites block the Li diffusion path. Moreover, the Ni^2+^ takes active Li^+^ place, which result in first‐cycle reversible capacity loss and poor layered structure stability.^12^, ^13^, ^14^, ^15^ In order to reduce antisite concentration, many approaches are utilized to improve the electrochemical performance, such as adjusting synthetic process, surface coating, and element doping. Zhou and co‐workers reduced the antisite of LiNi_0.8_Co_0.15_Al_0.05_O_2_ materials via control of oxygen flow rate during sintering.^16^ Zhang and co‐workers reported a novel multishelled Ni‐rich Li(Ni*_x_*Co*_y_*Mn*_z_*)O_2_ hollow fibers with low antisite as high‐performance cathode materials.^17^ On the contrary, few believe that antisite can play a good role in enhancing the stability of layered Ni‐rich cathode material.^18^, ^19^, ^20^, ^21^ The optimal degree of Ni^2+^ occupancy in the Li^+^ sites can improve the electrochemical performance of layered Li(Ni*_x_*Co*_y_*Mn*_z_*)O_2_ (NMC) materials and offer an electrostatic repulsion force to prevent more transition metal migration.^19^ Cho et al. found that the coating Ni‐based material with a thin antisite layer demonstrated an excellent cycling property under elevated temperature test, which is mainly ascribed to the unique pillar layer including moderate antisite concentration.^20^ Zheng et al. found that the Ni/Li antisite would decrease the thermal stability of the “Ni=Mn” group but benefit the thermal stability for “Ni‐rich” group.^21^ The antisite can be a powerful tool, or a double‐edged sward. However, as for this phenomenon, reasonable explanation has not been given. And the mechanism of introducing a favorable antisite remains ambiguous.

In this work, we first propose a mechanism of inducing the favorable antisite by bulk halogen‐doped to form the more stable local octahedron structure of halide (LOSH) [(Ni_2_Li_1_)‐halogen‐(Li_2_Ni_1_)]: halogen atom form three bonds with 2 Ni and 1 Li from the Ni layer, and form three bonds with 2 Li and 1 Ni from the Li layer in Ni‐rich layered cathode, combining NPD analysis with the first‐principles calculation. It is demonstrated that halogen substitution can easily induce antisite, especially for fluorine. NPD is an effective method to detect the presence and concentration of antisite; it is found that increasing F‐doped contents would promote the antisite concentration linearly and moderate antisite is beneficial for the structure stability, but excess will result in poor electronic conductivity, irreversible phase transformation, and destroy the spherical morphology of LNCM. Phase analysis and Mulliken charge showed that F substitution and antisite increase the spacing distance of (003) crystal plane in LOSH, which benefit Li^+^ migration. To confirm the effect of antisite on electrochemical/thermal properties, a type C80 microcalorimeter with 3D sensor was used to measure the heat released of different antisite ratio samples. As expected, the onset decomposition temperature of full lithiated cathode material increases by 48 °C. The cathode including moderate antisite (5.7%) displays super high cycling stability (94% retention at 1 C after 200 cycles under 25 °C), an outstanding high temperature cycling (a capacity of 153.4 mAh·g^−1^ at 1 C after 120 cycles under 55 °C), and improved rate capability (116.2 mAh·g^−1^ at 10 C) due to the new stronger LOSH. According to the theoretically calculated and experimental results, the proposed mechanism have been proven rational, which is a guideline to make use of antisite in Ni‐rich LNCM for the improvement of thermal stability, cycling performance, and rate capability for Ni‐rich LNCM cathode material.

It is well known that crystal structure with strong bonds will be more stable. As the atomic framework of LNCM consists of a close‐packed oxygen layer and interstitial transition metal ions (Ni, Co, Mn), if the stronger electronegative elements (X) are introduced into layered oxides to replace partial oxygen, the structure stability would be reinforced. The new forming X—Li (Ni, Co, Mn) bonds own higher bond strength than pristine O—Li (Ni, Co, Mn) bonds, resulting in a more stable layered oxides. However, interestingly it is found that the X plays a favorable role in the auxoaction for the formation of Li/Ni antisite; the reason is when X substitutes O site and the bond strength of X—Li is much greater than X—Ni, the adjacent Li^+^ and Ni^2+^ will exchange their sites to form a lower total energy and much stabler local octahedron structure. To verify this assumption of Li/Ni antisite induction mechanism, the F, Cl, Br, I, and S elements were selected to study here by using the first‐principles calculation based on density functional theory (DFT); their bond energy with Li, Ni, Co, and Mn are listed in Table S1 in the Supporting Information, and we can see that the F and Cl come closest to being a worthy replacement for the oxygen occupations(F‐Li:‐3.180 eV and Cl‐Li:‐2.107 eV are much lower than O‐Li:‐1.036 eV). Moreover, the bond strength of F—Li is nearly three times than that of F—Ni while the bond strength of O—Li is nearly equal to that of O—Ni, implying that the F is most likely to induce the Li/Ni antisite. The effect of F, Cl, Br, and I substitution on Li/Ni antisite formation energies are shown in **Figure** **1**a; the DFT calculation results indicate that both F and Cl can facilitate the antisite to form the most stable LOSH [(Ni_2_Li_1_)‐halogen‐(Li_2_Ni_1_)]: halogen atom form three bonds with 2 Ni and 1 Li from the Ni layer, and form three bonds with 2 Li and 1 Ni from the Li layer. And the formation energy of (Ni_2_Li_1_)‐F‐(Li_2_Ni_1_) in F‐LNCM, which is −0.221 eV, is the lowest one compared with other halogen‐doped compounds (Equations S3 and S4, Supporting Information). Thus, the Li/Ni antisite most easily occur in F‐LNCM structure, and this agrees with the above mechanism assumption. Figure 1b is the representative supercell of LNCM(811) sample, and other supercell of halogen‐doped LNMC samples based on LNCM(811) are built and calculated in our work, which are also the most energy‐stable structures.

**Figure 1:**
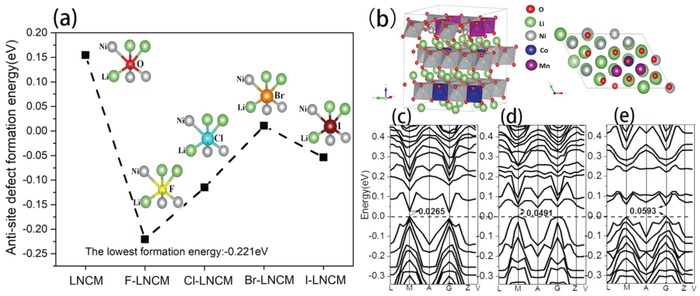
a) The effect of F, Cl, Br, and I substitution on the calculated antisite defect formation energies, the inset models are LOSH. b) The ground state crystal structure of LNCM(811) and calculated band structures of c) LNCM, d) 1% F‐LNCM, and e) 2% F‐LNCM.

Concisely tuning the Li/Ni antisite can be achieved by controlling the F substitution content. For the purpose of finding out the optimized doping, here we use the first‐principles calculation based on DFT to unveil the crystal and electronic structures of LNCM and F‐LNCM; the detailed calculation process is in Supporting Information. In the band structures (Figure 1c–e), there is a direct gap of ≈0.0265 eV with the conduction‐band minimum (cbm) and valence‐band maximum (vbm) at M‐point (Figure 1c). Therefore, it can be concluded that the pristine LNCM sample is a good electronic conductor. The band gap value of LiNiO_2_ computed by Ceder and co‐workers^22^ is 0.3 eV obtained with GGA+U, in which the introduction of Co and Mn can tune the band gap of LiNiO_2_, and improve the electronic conductivity. However, F enlarges the band gap compared with the undoped, and the gap values for 1% F‐LNCM, 2% F‐LNCM, and 3% F‐LNCM are 0.0491, 0.0593 (Figure 1d,e), and 0.0601 eV (Figure S1m, Supporting Information), respectively; thus the developing F content further increases the band gap. The widening of band gap indicates that the fluorination does decrease the electron transport, i.e., excess F would harm electronic conductivity for LNCM.

The total density of states (TDOS)^23^ and local density of states (LDOS) of F‐LNCM are different from those of the LNCM (Figure S1, Supporting Information), i.e., there is a new state near the Fermi level. From the LDOS of zero energy (Figure S1d–f, Supporting Information), the valence bond band near 0 eV for LNCM is mainly made up of O‐2p, Co‐3d, and Ni‐3d, followed by O‐2s and Mn‐3d, respectively. And the Li orbitals are far lower than fermi level and hence there is little free electron filling in Li orbitals, which indicate that Li atoms exist in LNCM‐layered structure in the form of ions (Figure S1g–l, Supporting Information). Moreover, the LDOS for 1% F‐LNCM and 2% F‐LNCM shows that the new state (from −7.5 eV to 2.5 eV) is mainly contributed by F‐2p orbital, and means the formation of F‐metal bonds. And the higher F content, the greater the contribution is helpful to reinforce framework. In addition, the introduction of F‐2p does not influence other element's contribution to the TDOS. From the above analysis, the bonds between O^2−^, F^−^, and transition metal (TM) ions are mainly constituted because of the strong interactions among them, and with increasing of F, the more F‐metal bonds form. Besides, moderate F doping can slightly raise voltage platform using DFT calculation (Equation S5 and Table S4, Supporting Information).

The calculations suggest that the optimized amount of F is 1% (molar ratio). When the ratio is over 1%, the band gap widens with the increasing amount of F dopant, and the wider band gap usually corresponds to the lower electronic conductivity. Thus, we deduce that excess F will against electronic conductivity. In addition, less F will not harm electronic conductivity, but may not induce moderate antisite.

LNCM, 1% F‐LNCM, and 2% F‐LNCM samples are synthesized and studied. The ratio among Li:Ni:Co:Mn as determined from inductively coupled plasma (ICP) is 1.05:0.85:0.075:0.075 (Table S3, Supporting Information), showing that the doped F hardly change sample's stoichiometric ratio. Both the Rietveld refinement of XRD (Figure S2, Supporting Information) and NPD (**Figure** **2**) are applied to confirm F site occupancy and Li/Ni antisite ratio quantitatively. XRD is less sensitive to the light atoms (e.g., Li^+^ ion; with small electron density) in comparison with NPD. Besides, XRD also encounters difficulty in differentiating the elements with similar electron densities (e.g., O vs F, and Mn vs Ni). While certain elements (or isotopes) could be differentiated by NPD due to the different scattering atomic factors, there has been no literature report on quantitative analysis for antisite via NPD.

**Figure 2:**
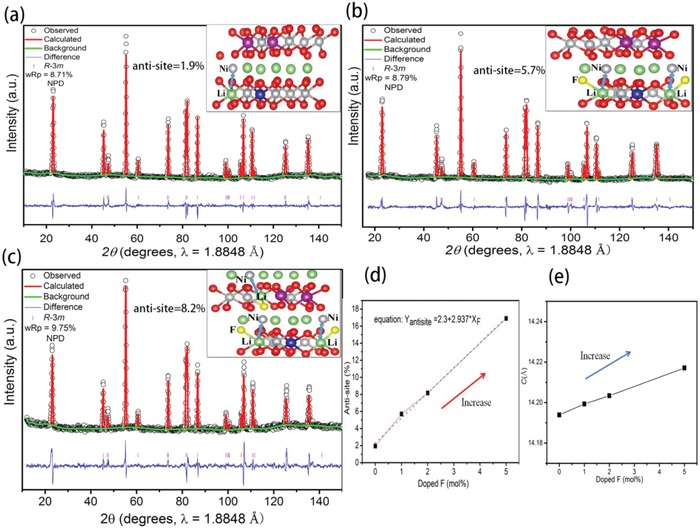
Neutron Rietveld refinement and the corresponding ground state crystal structures for a) LNCM, b) 1% F‐LNCM, and c) 2% F‐LNCM, respectively. d) The antisite concentration and e) changes in lattice parameter *c* with doped F content in the LNCM.

The Rietveld refinement of NPD patterns about LiNi_0.85_Mn_0.075_Co_0.075_O_2‐_ *_x_*F*_x_* are modeled by using trigonal phase with R3̅m space group. Figure 2a displays the refined NPD pattern of pristine material LNCM. Assuming Ni, Co, Mn atoms to be in the 3*b* sites, Li in the 3*a* sites, and O in the 6*c* sites in the trigonal phase, a good agreement can be obtained between data and the model. Site occupancy factors (SOF) of all atoms are refined by applying atomic position and displacement parameter constraints to the particular sites. The crystallographic information obtained from these refinements is presented in Table S6 in the Supporting Information. It shows that the antisite concentration for LNCM is about 1.9(2)%. Hence, antisite is verified to exist in the pristine material, and there is a very good match between experimental and calculated curves with a fitting parameter of *R*_wp_ = 8.71% (less than 10% is a good fitting degree). The Rietveld refinement profiles of F‐LNCM are shown in Figure 2b,c with low agreement values, 8.79% and 9.57%, respectively; these could be indexed to trigonal phase with R3̅m space group, similarly. The obtained crystallographic parameters are also presented in Table S6 in the Supporting Information. Firstly, the partial substitution of O with F in layered phase can result in good matching between data and model. It is found that a model with F (6*c*) can well describe the data. With the refined SOF for F (6*c*), a composition can be generated that is in accord with the nominal doping concentration in the synthetic process, indicating compositions with formula Li_1(2)_Ni_0.85(2)_Co_0.075_Mn_0.075_O_1.99_F_0.01_ and Li_1(2)_Ni_0.85(2)_Co_0.075_ Mn_0.075_O_1.98_F_0.02_, respectively. Interestingly, as the F content increases, the refinements show that more and more Li sites can be replaced by Ni; meanwhile, Li ion would also occupy vacancy of Ni. When doping mol amount of F are 1% and 2%, the antisite concentration are 5.7(2)% and 8.2(2)%, respectively. Both results support the same conclusion that the presence of F increases the antisite concentration. Quantitative relationship between the F‐doping content and antisite concentration shows a rather linear behavior (Figure 2d). Secondly, the lattice parameters of *a* and *c* change relatively little with F contents increasing, from 2.87799(1) to 2.87313(1) Å for the *a*, and from 14.1939(2) to 14.2034(6) Å for the *c*. The slow growth of *c* shows that there is a wider path for Li^+^ migration (Figure 2e). Thirdly, the NPD and XRD for 5% F‐LNCM (Figure S2d,e, Supporting Information) indicate that 5% F substitution inducing 16.7% antisite in LNCM sample contains about 17% rock‐salt phase that involves the redistribution of Li and Ni atoms across the Li layer and Ni layer (cation mixing);^24^ thus, excess F‐doped content can result in the irreversible new rock‐salt structure phase generation. In addition, there are no Mn‐Li and Co‐Li antisites in all investigated LiNi_0.85_Co_0.075_Mn_0.075_O_2‐_ *_x_*F*_x_* samples; the reason is that Co^3+^ ($t_{2g}^{6}e_{g}^{0}$) and Mn^4+^ ($t_{2g}^{3}e_{g}^{0}$) own the stable closed‐shell electronic configuration. In summary, NPD results provide accurate proof that F ions enter lattice to occupy the O sites and trigger the formation of Li/Ni antisite, and the antisite concentration increases with an increase in F‐doped amount.

The size and morphology of the samples are characterized by transmission electron microscope (TEM) and scanning electron microscopy (SEM). **Figure** **3**a shows a typical primary particle of the as‐synthesized F‐LNCM with a mean diameter of ≈0.5 µm. High resolution transmission electron microscope (HRTEM) analysis was also used to investigate the local structural change of F‐LNCM, and the direct distances were both ≈0.55 nm (Figure 3b), in a good agreement with (003) crystal facet of rhombohedral phase determined from NPD (Figure 2e). The observed *c* is larger compared with the pristine LNCM and could be helpful for the transport of Li^+^. A synergistic effect could be established through an F substitution–driven antisite. Mulliken charge is used to calculate atomic partial charge, and its variation trend can be applied for the atom charge distribution analysis.^25^ The calculated Mulliken charges of O in LNCM(PRI) and F‐LNCM(AS) are summarized in Figure 3c, such that the Mulliken charges for O (in the dashed box) in local layered structure of AS (below approximately −0.8 e per atom) become more negative compared with the pristine. The developing electrostatic repulsion force between oxygen layers increases the lattice parameter *c*. The reason is when F^−^ replaces the O^2−^ site, the local valence equilibrium state will be broken, and then the O atoms near F will get more negative and the Ni atoms near F get less positive to keep new valence equilibrium. In this case, there will be the mixing valence of cation that can facilitate Li^+^ transportation. The SEM images and energy dispersive X‐ray spectroscopy (EDS) mappings of a cross‐sectioned F‐LNCM particle manifest that the F atoms are distributed uniformly across the profile, together with the main elements, Ni and O (Figure 3d). This reveals that F has been successfully introduced both on surface and into the interior of secondary particle from the aspect of experiment. However, introduction of excess F will destroy the spherical morphology of LNCM (Figure S3, Supporting Information). As discussed above, the local structure change, that is the larger *d* spacings, are obtained due to the antisite and F substitution, which favor for Li^+^ migration. **Figure** **4** shows the approach on how to create the favorable antisite from the most stable LOSH. The influence of antisite on electrochemical performance and thermal behavior is further described below.

**Figure 3:**
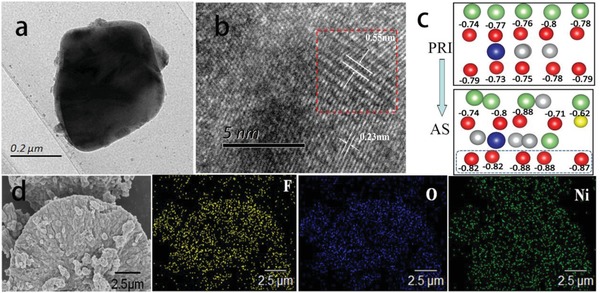
a) TEM image of F‐LNCM sample. b) HRTEM image and lattice fringes of F‐LNCM. c) The Mulliken charges of oxygen in LNCM(PRI) and F‐LNCM(AS). d) SEM image and EDS mappings of cross section for F‐LNCM.

**Figure 4:**
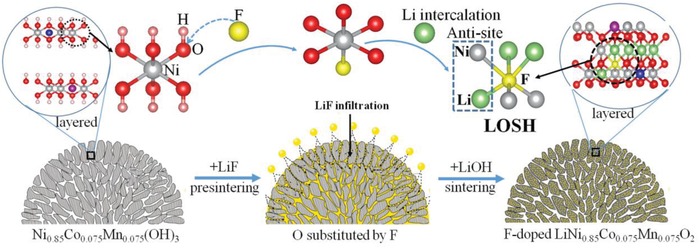
Schematic illustration of the preparation process of AS‐LNCM, indicating that the LiF compounds migrate into the particles of Ni_0.85_Co_0.075_Mn_0.075_(OH)_3_ and react with each other to facilitate O substituted by F; further sintering with LiOH generates the favorable antisite in the most stable LOSH to stabilize layered LNCM.

The initial charge and discharge curves in **Figure** **5**a show that the 1% F‐LNCM (5.7% AS) possesses higher charge/discharge voltage platform than LNCM(PRI), in line with previous calculation for the average delithiation potentials (Table S2, Supporting Information). The high temperature (55 °C) cycle performances at 1 C for PRI and 5.7% AS are displayed in Figure 5b. It shows that 5.7% AS owns an excellent high temperature cycling performance with a reversible capacity of 153.4 mAh·g^−1^ at 1 C for 120 cycles under 55 °C, and the capacity retention is 83.3%, while the capacity for PRI declines abruptly on the 85th cycle. From the inset SEM images of electrodes after cycling in Figure 5b, there is no trace of structural deterioration in 5.7% AS electrode compared with a large number of microcracks of PRI electrode, indicating a remarkable stable cycling process of 5.7% AS. From 1st cycle to 30th cycle (Figure 5c), it is found that the capacity offers slight performance improvement due to the fact that high temperature reduces activation time. From 1st to 120th cycle, there is still an irreversible capacity of 153.4 mAh·g^−1^ with a small polarization. Besides, the 5.7% AS cathode performs an exceptionally excellent cyclability at room temperature (Figure 5d); more than 94% of initial capacity was maintained after extended 200 cycles. These outstanding cycling performances are attributed to the favorable antisite of the LOSH that is the most stable local structure. Figure 5e compares the rate capabilities of PRI, 5.7% AS, and 8.2% AS at different rates. When charge–discharge rate is 0.1 C (1 C = 200 mAh·g^−1^), it is clear that the initial discharge capacity (PRI: 202.9 mAh·g^−1^, 5.7% AS: 195.4 mAh·g^−1^, 8.2% AS: 186 mAh·g^−1^) is inversely proportionate to antisite concentration in Figure S4 in the Supporting Information. This first‐cycle capacity decay is ascribed to the Li occupied by Ni, resulting in partial loss of activated Li. The discharge capacities of PRI and 8.2% AS decrease significantly from 0.1 to 10 C, whereas the 5.7% AS decrease much more slowly at the same rate. Even at 10 C, that is a time of 6 min to fully charge/discharge the cathodes, and the 5.7% AS still maintains a discharge capacity of 116.2 mAh·g^−1^, much higher than that of PRI and 8.2% AS (≈98.6 and 93.3 mAh·g^−1^, respectively). When the rate is restored to 0.1 C, the reversible capacity of 5.7% AS is also recovered to ≈189 mAh·g^−1^, implying the excellent tolerance to the rapid Li ion intercalation/deintercalation. Further increasing antisite content occurs at the expense of rate performance, indicating that the excess antisite would harm the kinetics of LNCM. And the electrochemical impedance spectrum (EIS) results of 5.7% AS and 8.2% AS electrodes show the smaller *R*_ct_ and *R*_cei_ (resistance of cathode electrolyte interphase) values, and larger *D*_Li+_ than the PRI, demonstrating that moderate antisite can enhance the kinetic activity and particle interfacial film stability of Ni‐rich cathode (Figure S5 and Table S7, Supporting Information; the *D*_Li+_ calculations are in Supporting Information). Figure 5f,g displays the charge/discharge voltage profiles of the PRI and 5.7% AS cathodes from 0.1 to 10 C, and the 5.7% AS exhibits a small polarization compared with the PRI. This high rate capability of 5.7% AS shows an excellent kinetic behavior, which is ascribed to the improved Li^+^ diffusion originated from the developing electrostatic repulsion force between oxygen layers that increase the lattice parameter *c*, rapid charge transfer, and stable cathode electrolyte interface (CEI) film.

**Figure 5:**
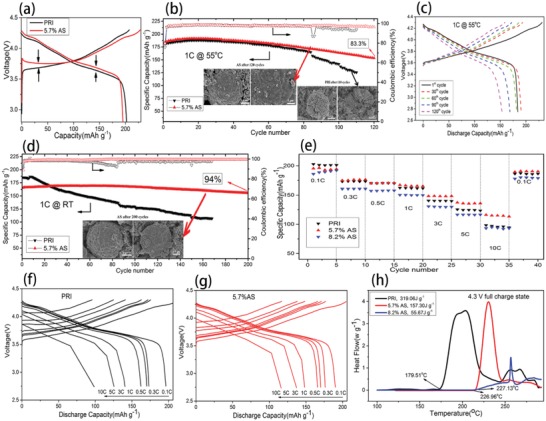
a) Initial charge and discharge curve of LNCM(PRI) and 1% F‐LNCM (5.7% AS) cathodes at a rate of 0.1 C. b) High‐temperature (55 °C) cycling performance over 120 cycles at a rate of 1 C; the inset SEM images are cathode after cycling. c) The charge and discharge curves of 5.7% AS cathodes at 1 C under 55 °C. d) Room‐temperature cycling performance over 200 cycles at a rate of 1 C. e) Rate capability of PRI, 5.7% AS, and 8.2% AS cathodes. 0.1–10 C rate charge/discharge curves for f) PRI and g) 5.7% AS. h) C80 profiles of delithiated PRI, 5.7% AS, and 8.2% AS cathodes that were charged to 4.3 V in advanced.

The type C80 microcalorimeter was applied to measure the thermal properties of PRI, 5.7% AS, and 8.2% AS electrodes at fully delithiated state (Figure 5h). Obviously with lower decomposition temperature and heat generation, which, for PRI, 5.7% AS, and 8.2% AS, are 179.51 °C and 319.06 J·g^−1^, 226.96 °C and 157.30 J·g^−1^, and 227.13 °C and 55.67 J·g^−1^, respectively. As mentioned, the heat originates from structure phase transition and O_2_ release during charging. The C80 results indicate that the favorable antisite enhances the onset temperature for two separate reasons: 1) the Ni migrates into Li layer to form the most stable LOSH^20^; 2) the antisite prevents more Ni to migrate into Li layers restraining the phase change of layered structure. In addition, the replacement of O by F reduces the possible oxidation of active O^2−^ further to O_2_. The peaks under three antisite ratios represent phase conversion temperature corresponding to the transformations to spinel and rock‐salt structures and concomitant O_2_ release. The presence of antisite as demonstrated by NPD analysis can effectively raise the phase conversion temperature because of strengthened LOSH, which is proportional to antisite ratio.

In summary, according to the first‐principles calculation and NPD results, we have proposed a defect inducing the favorable antisite mechanism; the oxygen substituted by halogen promote the neighboring Li and Ni atoms to exchange their sites to form the more stable LOSH. Based on the mechanism, we successfully synthesized the F‐doped LNCM samples containing varying contents of antisite. The NPD and XRD analysis demonstrated that the antisite ratio can be precisely regulated by adjusting content of F doped within limits, but excess antisite lead to an irreversible phase transformation from layered to rock‐salt structure reducing the specific capacity. The moderate 5.7% antisite in LNCM showed an excellent cycling performance, and especially outstanding under the condition of high temperature (55 °C). The cycling performance and decomposition onset temperature of this cathode are remarkably stable and higher compared with pristine LNCM, and these wonderful performances are attributed to the favorable antisite in LOSH because it owns the lowest formation energy and the most stable local structure to keep the structural integrity of LNCM particles after long cycling. Besides, the valence distribution from the synergistic effect combining antisite and F substitution in LOSH causes the larger *c* and thus allows fast Li^+^ migration in Li layer, which improves rate capability. Our studies put forward a novel mechanism on antisite and a new approach to create the favorable antisite in Ni‐rich cathode materials; these new insights provide design principle of antisite for high electrochemical and thermal performance layered and olivine‐type cathode materials for Li‐ion/Na‐ion batteries.

## Experimental Section

*Computational Methodology*: DFT calculations were executed with pseudopotentials established by the projector‐augmented wave (PAW) method and the Perdew–Burke–Ernzerhof (PBE) exchange‐correlation functional using the Vienna ab initio simulation package (VASP). The computational details are follows. The k‐mesh was G‐centered. The Hubbard‐type correction U for the Mn‐3d, Ni‐3d, Co‐3d states were taken into consideration and the Hubbard *U* values (*U–J*) were 6.5, 4.9, and 4.9 eV used in Ni, Co, and Mn ions, respectively. The plane‐wave cutoff was set to 520 eV. The primitive cell of R‐3m Li_3_Ni_3_O_6_ was relaxed within 5 × 7 × 3 k‐meshes. For the F‐doped structure, R‐3m Li_18_Ni_14_Co_2_Mn_2_O_36‐_ *_x_*F*_x_* (*X* = 1, 2), a (3 × 2 × 1) supercell (18LiMO_2_) was used as the calculation model and only the G‐point was considered. Firstly, building all the possible substitution patterns of Ni swapped with Co and Mn in Li_18_Ni_18_O_36_ were considered (66 forms) and the structure of the lowest energy state was picked out. Secondly, all the possible models of fluorinated oxygen (36 forms) in Li_18_Ni_14_Co_2_Mn_2_O_36_ were structured and the ground states were obtained. Then, the structures of Li_18_Ni_14_Co_2_Mn_2_O_36_ and Li_18_Ni_14_Co_2_Mn_2_O_36‐_ *_x_*F*_x_* were used in the electronic structure, charge transfer, charge compensation calculations. All the computations were carried on at National Supercomputing Center in Shenzhen.

*Material Synthesis*: The synthesis of Ni‐rich LiNi_0.85_Mn_0.075_Co_0.075_O_2‐_ *_x_*F*_x_* (*X* = 0.01, 0.02, 0.05) powders were prepared by a hydroxide route using transition‐metal hydroxide, lithium hydroxide, and lithium fluoride as starting materials. The precursor Ni_0.85_Mn_0.075_Co_0.075_(OH)_3_ was fabricated by a coprecipitation method with raw materials consisting of NiSO_4_·6H_2_O, CoSO_4_·7H_2_O, MnSO_4_·5H_2_O, NH_4_OH, NaOH. And then, the precursor powders were obtained by filtering, washing, and drying in a vacuum oven at 110 °C overnight. Finally, the obtained Ni0_0.85_Mn_0.075_Co_0.075_(OH)_3_ was mixed with LiF and LiOH (F/(Ni + Co + Mn) = 0.01, 0.02, 0.05 in a molar ratio and Li/(Ni + Co + Mn) = 1.05), and the mixtures were calcined at 550 °C for 5 h and 750 °C for 12 h in the oxygen atmosphere, respectively.

*Material Characterization*: The chemical compositions of the samples were analyzed by inductively coupled plasma atomic emission spectrometry (ICP‐AES). The crystal structure of all the LiNi_0.85_Mn_0.075_Co_0.075_O_2_ and LiNi_0.85_Mn_0.075_Co_0.075_O_2_F*_x_* (*X* = 0, 0.01, 0.02) were measured by XRD (Rigaku UltimaIV‐185) with Cu Kα radiation at a scan rate of 8°*2θ* min^−1^. Neutron diffraction patterns were carried out at ambient temperature using high resolution neutron powder diffactometer (HRND) at China Mianyang Research Reactor (CMRR), which operates with a monochromatic X‐ray of λ = 1.8848 Å. The scans were collected between 15° and 150° (2θ) at a step size of 0.06°. The X‐ray and neutron datasets were refined by the conventional Rietveld method using the General Structure Analysis System (GSAS) package with the graphical user interface (EXPGUI). The background, scale factor, zero (for X‐ray), absorption (for neutron), cell parameters, atomic positions, thermal parameters, and profile coefficients for Pseudo‐Voigt/Finger, Cox, and Jephcoat (FCJ) Asymmetric peak shape function were refined until the convergence was achieved.^26^ The morphologies of materials were obtained via using SEM (JSM 6340F, JEOL).

*Electrochemical Measurement*: The cathode electrodes consisted of the prepared powder: carbon black:poly(vinylidene fluoride) (PVDF) binder = 8:1:1 (by weight). Coin cell (Model CR2025) was used as test cell, while metal lithium foil was used as a counter electrode. The cyclic voltammetry of cathode electrodes were carried out in a three‐chamber cell with two lithium foils as counter electrode and reference electrode separately, on a CHI660 A electrochemical workstation in the potential range: 2.7–4.3 V (vs Li/Li^+^). The cutoff potentials for charge (intercalation of lithium) and discharge (deintercalation of lithium) processes (by LAND battery testing system) were 2.7 and 4.3 V (vs Li/Li^+^), respectively. The specific capacities presented in this article are based on per gram LiNi_0.85_Mn_0.075_Co_0.075_O_2_F*_x_* for cathode electrode.

*Thermal Evaluation of Full Charge State*: For the type C80 microcalorimeter (Setaram Co.) with 3D sensor analysis, 2025 coin type cells were charged to 4.3 V and opened in an Ar‐filled dry room. The electrodes were washed several times in dimethyl carbonate, and then the cathode materials were exfoliated from the current collector. A stainless steel cylinder with a gold‐plated copper seal was used to collect 12–20 mg samples. Measurements were performed using a C80 microcalorimeter at a temperature scan rate of 0.01 °C min^−1^.

## Conflict of Interest

The authors declare no conflict of interest.

## Supporting information

Supplementary
