# Supplementary material for: Inducing Favorable Cation Antisite by Doping Halogen in Ni‐Rich Layered Cathode with Ultrahigh Stability
Source: Adv Sci (Weinh). 2018 Dec 12;6(4):1801406. doi: 10.1002/advs.201801406 (PMC6382300; doi:10.1002/advs.201801406)
Supplement: Supplementary file 1 — Supplementary [file ADVS-6-1801406-s001.pdf]

Copyright WILEY-VCH Verlag GmbH & Co. KGaA, 69469 Weinheim, Germany, 2018.

ADVANCED SCIENCE Open Access

Supporting Information

for *Adv. Sci.,* DOI: 10.1002/advs.201801406

Inducing Favorable Cation Antisite by Doping Halogen in Ni-Rich Layered Cathode with Ultrahigh Stability

Chunli Li, Wang Hay Kan, Huilin Xie, Ying Jiang, Zhikun Zhao, Chenyou Zhu, Yuanhua Xia, Jie Zhang, Kang Xu, Daobin Mu, and Feng Wu**

((Supporting Information can be included here using this template))

Copyright WILEY-VCH Verlag GmbH & Co. KGaA, 69469 Weinheim, Germany, 2016.

Supporting Information

Inducing Favorable Cation Anti-site by Doping Halogen in Ni-rich Layered Cathode with Ultra-High Stability

Chunli Li, Wang Hay Kan, Huilin Xie, Ying Jiang, Zhikun Zhao, Chenyou Zhu, Yuanhua Xia, Jie Zhang, Kang Xu, Daobin Mu,* and Feng Wu*

Using bond energy/strength can acquire the information of local structural stability in a new material. Because, the bond energy for X-Li (Ni, Co, Mn) bonds are obtained by calculating the formation energy of solids state materials, they are built as supercell and calculated through DFT computation. The calculation method is as follows: ① to calculate the formation energy of X-M (X:O, F, Cl, Br, I, S; M: Li, Ni, Co, Mn) solids state materials by using the formula,

Equation S1. $$\Delta E_f(X-M) = E(X-M) - E(X) - E(M) \quad (1)$$

Where Δ_f E(X-M) is the formation energy for X-M (The X-M represent the crystalline compounds consisting of halogen and metallic elements, in which M is metallic element and X is halogen element.) solid supercell, E(X-M), E(X) and E(M) are the energies of the X-M solid supercell, pure X and pure M, respectively. Because the value of bond energy is approximately equal to the bond enthalpy, thus we apply the bond enthalpy calculation method to obtain the value of bond energy. That is, bond breaking enthalpy minus bonding enthalpy is the bond enthalpy. Thus, herein, we express the value for bond enthalpy as the bond energy.

② to calculate the formation energy of X-M bonds:

Equation S2. $$\Delta E_f(bond) = \Delta E_f(X-M) \div \mathrm{n} \quad (2)$$

Where Δ_f E(bond) is the average formation energy for X-M bonds in solid supercell, n is the number of bonds in the supercell. All the bond formation energies between X (O, F, Cl, Br, I, and S) elements and metals (Li, Ni, Co, and Mn) are listed in Table S1.

Table S1. Bond formation energies between X (O, F, Cl, Br, I, and S) elements and metals (Li, Ni, Co, and Mn)

Bond Formation(eV)	Li	Ni	Co	Mn
O	-1.0355	-0.9340	-1.0628	-0.9055
F	-3.1800	-1.0650	-0.7230	-0.5870
Cl	-2.1070	-0.4870	-0.4650	-0.3700
Br	-1.5740	-0.3640	-0.145	-0.4085
I	-1.1990	-0.0505	0.0125	-0.2285
S	-0.7425	-0.3470	-0.5150	-0.4950

To build accurate models of LNCM and halogen-doped LNCM, $LiNiO_2$ that owns a space group with rhombohedral symmetry (R-3m) based on α-$NaFeO_2$ was used as the building-block, with an enlarged (3x2x1) supercell of $Li_{18}Ni_{18}O_{36}$. In order to calculate the arrangement of transition metals (Co, Mn) at relative accuracy so that $Li_{18}Ni_{14}Co_2Mn_2O_{36}$ model could be constructed, we substituted each site of Ni with Co or Mn, with a total of 66 structure types. In these configurations where Co and Co atoms are located in neighboring layers while Mn and Mn atoms positioned neighboring each other shows the lowest total energy (Figure 1b). After that, on the basis of the LNCM parent model, the model oxygen atom substituted by fluorine atom can be made. halogen-doped LNCM can own thousands of possible variation due to micro-content of fluorine. To simplify the large amount of calculation, first halogen substitutions were made 36 times and the optimum structure can be selected; second halogen substitutions were carried on at sites adjacent to the first halogen and so on.

The formation energy ($\Delta_f E$) of F-doped is computed as follows.

Equation S3. $$\Delta_f E^{O,X} = E(LiMO_2^{O,X}) + \mu(O) - E(LiMO_2^{P}) - \mu(X) \quad (3)$$

Where $\Delta_f E^{O,x}$ is the formation energy when a X atom is placed at an oxygen-site, $E(LiMO_2^{O,x})$ and $E(LiMO_2^{P})$ are the energies of the halogen-doped and pristine

LNCM, μ(O) and μ(X) are the atomic chemical potentials of the O and halogen, respectively. Based on the $Li_{18}Ni_{14}Co_2Mn_2O_{36}$ supercell model, each site of O was substituted by F, with a total of 36 structure types. The formation energy of these 36 structure types were calculated by using DFT, shown in Table S2. The 17th type structure has the lowest formation energy (-0.21014 eV), thus it was selected the optimized structure for $Li_{18}Ni_{14}Co_2Mn_2O_{35}F$.

Table S2. The formation energies of 36 structure types for $Li_{18}Ni_{14}Co_2Mn_2O_{35}F$

1~12	$\Delta_f E^{O,F}$(eV)	13~24	$\Delta_f E^{O,F}$(eV)	25~36	$\Delta_f E^{O,F}$(eV)
1	49373.29	13	-0.01467	25	0.03313
2	0.31773	14	0.25479	26	-0.07934
3	0.94458	15	0.35605	27	-0.03417
4	0.55199	16	0.00049	28	-0.10288
5	0.15926	17	-0.21014	29	0.13493
6	0.34146	18	-0.15197	30	0.31277
7	0.38485	19	-0.15116	31	0.06091
8	0.10561	20	0.20977	32	-0.12312
9	0.3936	21	0.24652	33	0.35116
10	0.94409	22	-0.02796	34	0.00405
11	-0.13742	23	0.01978	35	-0.09927
12	0.31595	24	-0.11706	36	0.4746

The anti-site formation energy is computed as follows.

Equation S4. $$\Delta_f E(\text{anti-site}) = -E(\text{perfect}) + E(\text{defective}) \quad (4)$$

Where E(perfect) is the total lattice energy of the perfect halogen-doped and pristine LNCM, E(defective) is the total lattice energy of the halogen-doped and pristine LNCM including anti-site. The formation energies of anti-site were calculated by using DFT, shown in **Table S3**.

Table S3. The calculated anti-site defect formation energies in LNCM, F-LNCM, Cl-LNCM, Br-LNCM and I-LNCM

	LNCM	F-LNCM	Cl-LNCM	Br-LNCM	I-LNCM
Δ_fE(anti-site)/eV	0.15449	-0.22065	-0.11496	0.01061	-0.05384

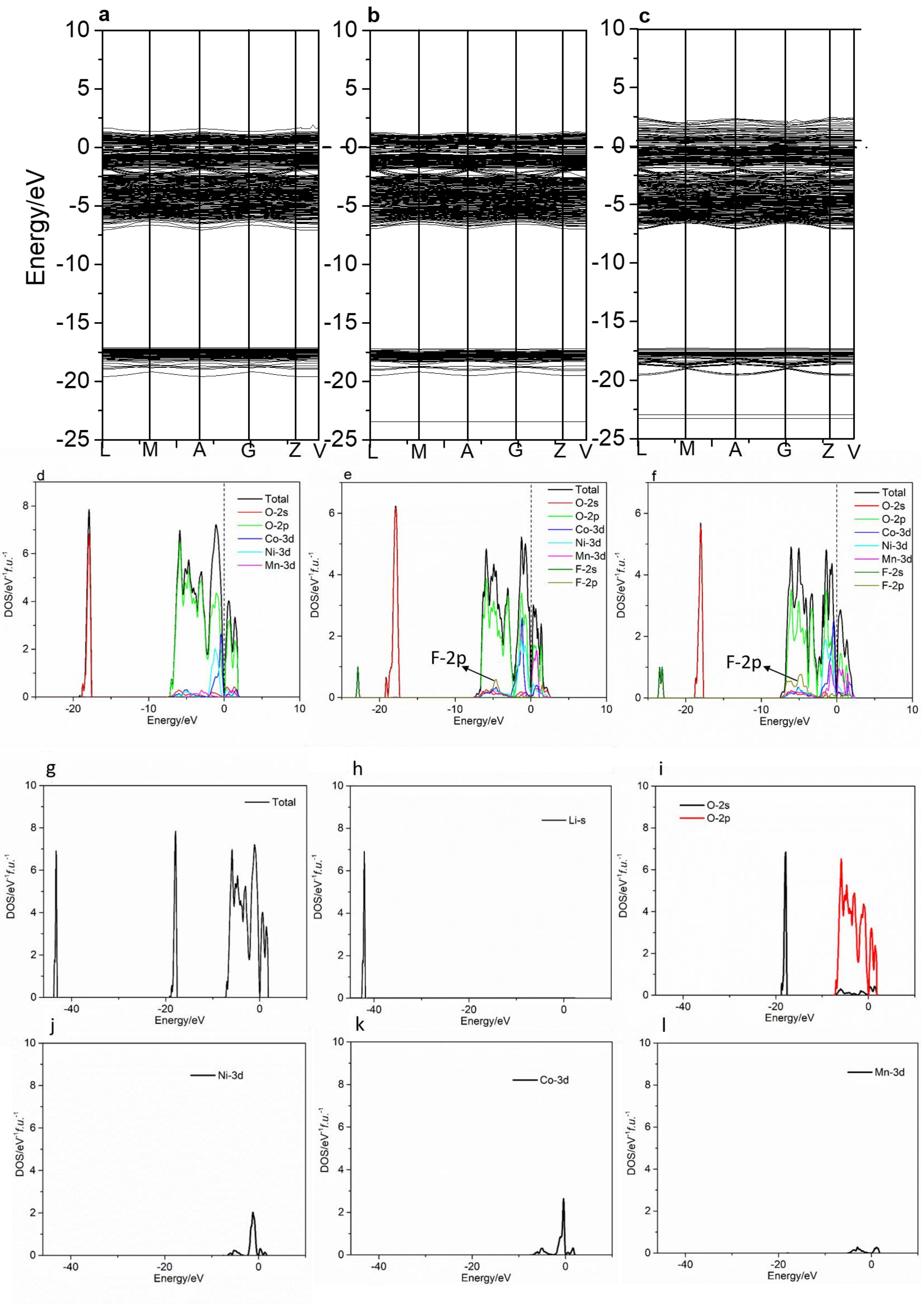
a
b
c
Energy/eV
L M A G Z V
d
e
f
Total
O-2s
O-2p
Co-3d
Ni-3d
Mn-3d
F-2s
F-2p
DOS/eV⁻¹f.u.⁻¹
Energy/eV
g
h
i
j
k
l
Li-s

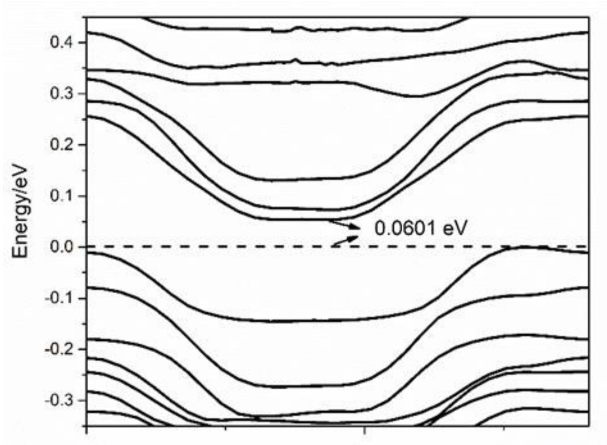

Figure S1. Calculated band structure from -25 eV to 10 ev of (a) LNCM, (b) 1%F-LNCM and (c) 2%F-LNCM; Calculated total density of states (TDOS) and local density of states (LDOS) of (d) LNCM, (e) 1%F-LNCM and (f) 2%F-LNCM. The zero energy is set to the Fermi level. Calculated total density of states of LNCM (g) and partial density of states for Li (h), O (i), Ni (j), Co (k) and Mn (l). (m) calculated band structure for 3%F-LNCM.

Figure S1(g-i) shows the calculated total density of states of Ni-rich LNCM and partial density of states for Li, O, Ni, Co and Mn. The first valence band appears near -42.5 eV (**Figure S1g**), through the comparison from Figure 2b~2f, we know that the first valence band is only made up of Li-s, that is far away from fermi energy (0 eV), means there are only valence electrons filling in Li orbitals, that is inactive. The second valence band near -18 eV is composed of O-2s. More importantly, the electronic states near 0 eV and conduction band (it is the energy space formed by free electrons and located at higher than 0 eV) are mainly made up of O-2s, O-2p, Ni-3d, Mn-3d and Co-3d. Which indicate there are a lot of active free electrons filling in O-2s, O-2p, Ni-3d, Mn-3d and Co-3d orbitals, and thus there mainly existed a strong bonding interaction between O and transition metal (Ni, Co and Mn) in Ni-rich LNCM crystal. To sum up, first, because Li orbitals are located in valence band and thus there is no free electron to interact with that of other elements; second, Li has a smaller contribution to the total density of states for Ni-rich LNCM. Therefore, Li atoms exist in LNCM layered structure in the form of ions.

The average delithiation potentials of F-LNCM are obtained by applying **Equation S5**, and the calculation results given by GGA+U method are listed in **Table S2**. Where V_{ave} represents the average delithiation potential, $E(MO_2)$ is the total energy of delithiated structures obtained by removing all Li atoms from the $LiMO_2$ structures. The V_{ave} is an extremely important parameter for energy densities of lithium-ion battery that is equal to the product between average delithiation potentials and specific capacities. **Table S2** shows the calculated average delithiation potentials at different F doping levels, the V_{ave} increases as the F doping content increases, which derives from that F changes the electronic structure and total energy of LNCM, and consequently leads to the average delithiation potential is raised from 3.91V(the result is consistent with Ceder[1]) to 3.96V. The doped F contributes to enhance V_{ave} within a certain content limit, therefore F doping is a good way to raise voltage platform.

Equation S5.

$$V_{ave} = -\frac{E(MO_2) - E(LiMO_2^p) + E(Li)}{-e} \quad (5)$$

Table S4. The calculated average delithiation potentials of F_xLNCM (X=0, 0.01 vs Li).

X_F	E(before) (eV f.u.-1)	E(after) (eV f.u.-1)	Delithiation Voltage(Vave)	Band Gap(eV)
0	-2336.89	-2131.42	3.91	0.0265
0.01	-2349.34	-2143.82	3.96	0.0491

Table S5. Results of the ICP analysis for $LiNi_{0.85}Co_{0.075}Mn_{0.075}O_{2-x}F_x$.

composition	Mole fraction of materials			
	Ni	Co	Mn	Li
X_F=0	0.847	0.075	0.073	1.054
X_F=0.01	0.849	0.074	0.076	1.055
X_F=0.02	0.842	0.082	0.081	1.000

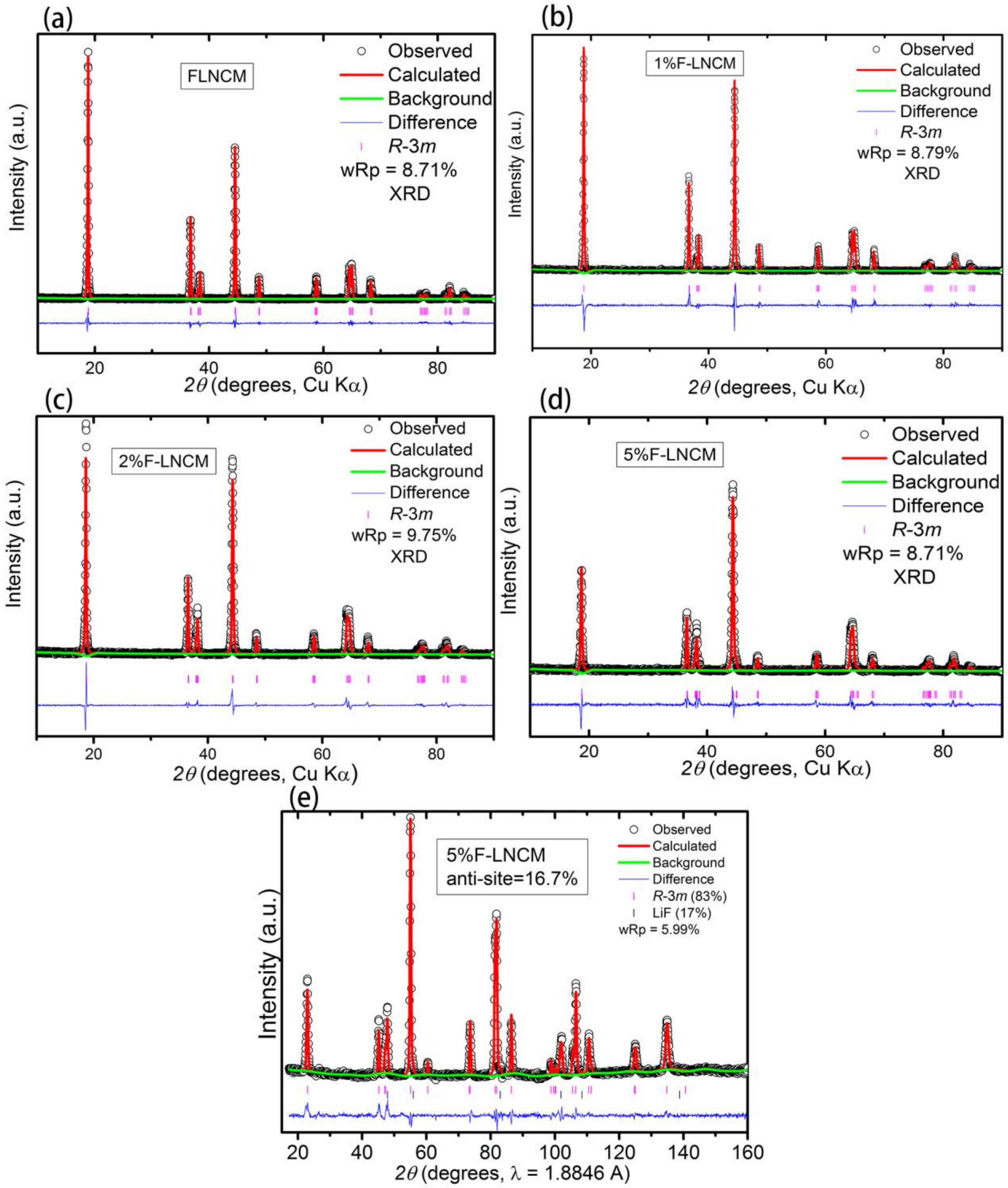

Figure S2. XRD Rietveld refinement for (a)LNCM, (b)1%F-LNCM, (c)2%F-LNCM and (d)5%F-LNCM respectively. NPD Rietveld refinement for (e)5%F-LNCM.

Table S6. Crystallographic details of the $LiNi_{0.85}Mn_{0.075}Co_{0.075}O_{2-x}F_x$ obtained from joint Rietveld analysis using three NPD datasets.

atom	site	x	y	z	SOF	100*Uiso (Å2)
LiNi0.85Mn0.075Co0.075O2 Lattice Parameters: a= 2.87799(1)Å; c = 14.1939(2)Å; Rwp = 8.71%						
Li	3a	0	0	0	0.981(2)	1.2(2)
Ni	3a	0	0	0	0.019(2)	
Ni	3b	0	0	0.5	0.831(2)	0.94(4)
Mn	3b	0	0	0.5	0.075	
Co	3b	0	0	0.5	0.075	
Li	3b	0	0	0.5	0.019(2)	
O	6c	0	0	0.2412(1)	1	0.82(5)
LiNi0.85Mn0.075Co0.075O1.99F0.01 Lattice Parameters: a=2.87553(1)Å; c = 14.1993(4)Å; Rwp = 8.79%						
Li	3a	0	0	0	0.943(2)	1.0(2)
Ni	3a	0	0	0	0.057(2)	
Ni	3b	0	0	0.5	0.793(2)	0.23(5)
Mn	3b	0	0	0.5	0.075	
Co	3b	0	0	0.5	0.075	
Li	3b	0	0	0.5	0.057(2)	
O	6c	0	0	0.2423(1)	0.995	1.41(8)
F	6c	0	0	0.2423(1)	0.005	
LiNi0.85Mn0.075Co0.075O1.98F0.02 Lattice Parameters: a=2.87313(1)Å; c = 14.2034(6) Å; Rwp = 9.75%						
Li	3a	0	0	0	0.918(2)	0.69(6)
Ni	3a	0	0	0	0.082(2)	
Ni	3b	0	0	0.5	0.768(2)	0.1(1)
Mn	3b	0	0	0.5	0.075	
Co	3b	0	0	0.5	0.075	
Li	3b	0	0	0.5	0.082(2)	
O	6c	0	0	0.2418(2)	0.99	1.6(1)
F	6c	0	0	0.2418(2)	0.01	
LiNi0.85Mn0.075Co0.075O1.98F0.05 Lattice Parameters: a=2.88038(1)Å; c = 14.2092(6) Å; Rwp = 5.99%						
Li	3a	0	0	0	0.832(2)	0.21(2)
Ni	3a	0	0	0	0.167(2)	
Ni	3b	0	0	0.5	0.682(2)	0.21(1)
Mn	3b	0	0	0.5	0.075	
Co	3b	0	0	0.5	0.075	
Li	3b	0	0	0.5	0.167(2)	
O	6c	0	0	0.2439(2)	0.95	1.6(1)
F	6c	0	0	0.2439(2)	0.05	

SOF = site occupancy factor and Uiso = isotropic atomic displacement parameter.

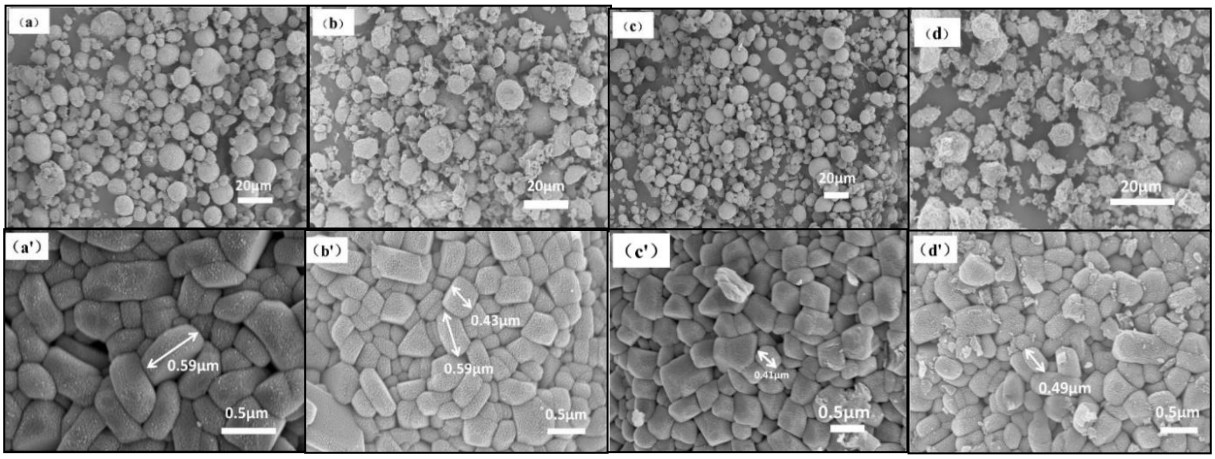

Figure S3. SEM images of (a) LNCM,(b) 1%F-LNCM,(c) 2%F-LNCM,(d) 5%F-LNCM.

The SEM images of pristine $LiNi_{0.85}Co_{0.075}Mn_{0.075}O_2$ and F-doped $LiNi_{0.85}Co_{0.075}Mn_{0.075}O_2$ display in figure S3a-d'. It is transparent that the increased fluorine content have a significant impact on the morphology of LNCM particals. Which specific in two fronts, for secondary particle, the higher the content of introduced fluorine, the more incomplete the sphere is; and there are many chippings with increased fluorine. With regard to the primary particle, the primary particle size changed a little. When F content is 1%, there is no obvious change, however, when F content exceeds 2%, the average size and edge of the primary particles are becoming smaller and blur, respectively. In addition, many ~80nm fragments adhesion to the primary particle are discovered.

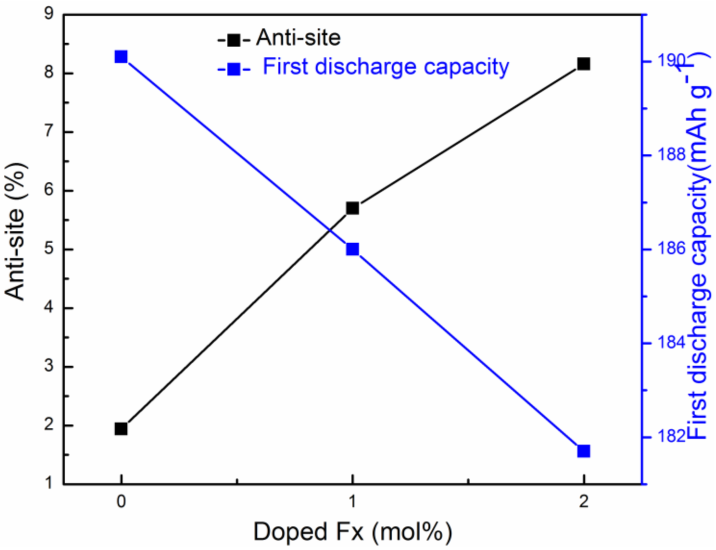

Figure S4. The relationship between anti-site and first discharge capacity.

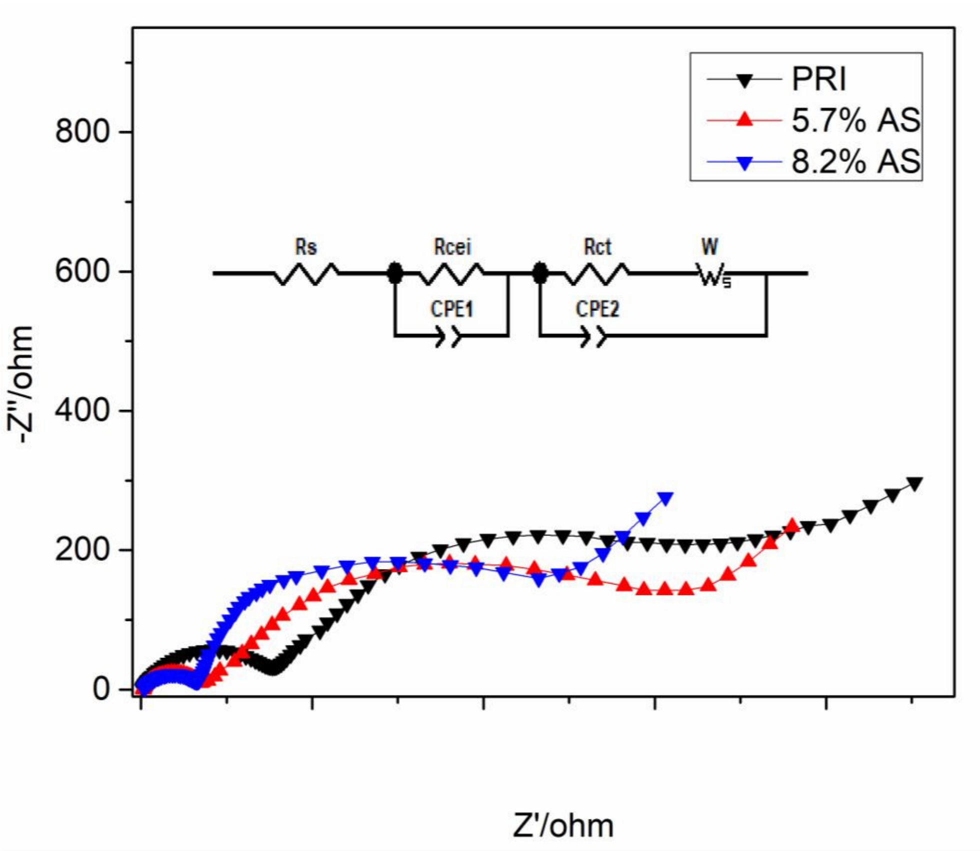

Figure S5. Comparison of Nyquist plots of PRI, 5.7% AS and 8.2% AS electrodes at fresh state (3V) (the inset is equivalent circuit).

Table S7. The calculation results of resistances for PRI, 5.7% AS and 8.2% AS electrodes.

	R_{cei} (ohm)	R_{ct} (ohm)
PRI	161.4	757.8
5.7% AS	66.2	505.6
8.2% AS	66.6	443

The **Figure S5** displays the EIS results of PRI, 5.7% AS and 8.2% AS electrodes at fresh state (3V). The inset is equivalent circuit, R_s, R_{cei} and R_{ct} represent the ohmic resistance of the electrolyte between the working electrode and the reference electrode, cathode electrolyte interphase film and charge transfer, respectively. The calculated fitting values of R_{cei} and R_{ct} are presented in **Table S7**. The R_{ct} values of 5.7% AS and 8.2% AS are about 505.6 and 443 Ω, which are smaller than 757.8 Ω of PRI, indicating that moderate anti-site can improve kinetic activity of Ni-rich cathode. And, the R_{cei} for 5.7% AS (66.2 Ω) and 8.2% AS (66.6 Ω) clearly decrease compared with the PRI (161.4 Ω), the reason is that moderate anti-site may

suppress the decomposition of electrolyte, and the stable CEI film with lower resistance forms on the Ni-rich material surface. Thus, the EIS demonstrate that moderate anti-site enhance the kinetic activity and particle interfacial film stability of Ni-rich cathode. According to EIS results (**Figure S5**), the Li^+ diffusion coefficient in these three samples can be obtained from an analysis of the Warburg impedance.

Equation S6. $$-\mathrm{Im}(Z_w) = B\omega^{-1/2} \quad (6)$$

Equation S7. $$D_{\mathrm{Li}^+} = 0.5\left[\frac{V_m}{FSB}\left(-\frac{dE}{dx}\right)\right]^2 \quad (7)$$

Where ω is the angular frequency, and B is the Warburg coefficient (0.02837 for PRI, 0.01002 for 5.7% AS and 0.01031 for 8.2% AS). V_m is molar volume of the samples (33.85cm^3/mol for PRI, 33.89cm^3/mol for 5.7% AS and 33.94cm^3/mol for 8.2% AS, these values are obtained by NPD refinement), S is the apparent surface area of the electrode (~1.33cm^2, it is smaller than real surface area, because the sample in our manuscript is not a film electrode), F is 96500 C/mol, dE/dx is the slope of the open-circuit potential vs. Li-ion concentration x at each x value(-0.0072 for PRI, -0.0037 for 5.7% AS and -0.0036 for 8.2% AS). For pure PRI sample, the D_{Li+} is about 2.24×10^{-9} cm^2/S, and for the 5.7% AS and 8.2% AS samples, the D_{Li+} are estimated to be 4.26~4.75×10^{-9} cm^2/S. Because of the electrode surface area, the calculated D_{Li+} will be little bit larger than true values. But, the relative values of the 3 samples are accurate and meaningful, that indicate moderate anti-site promote the Li^+ diffusion, the possible reason is the developing electrostatic repulsion force bewteen oxygen layers increase the lattice parameter c.

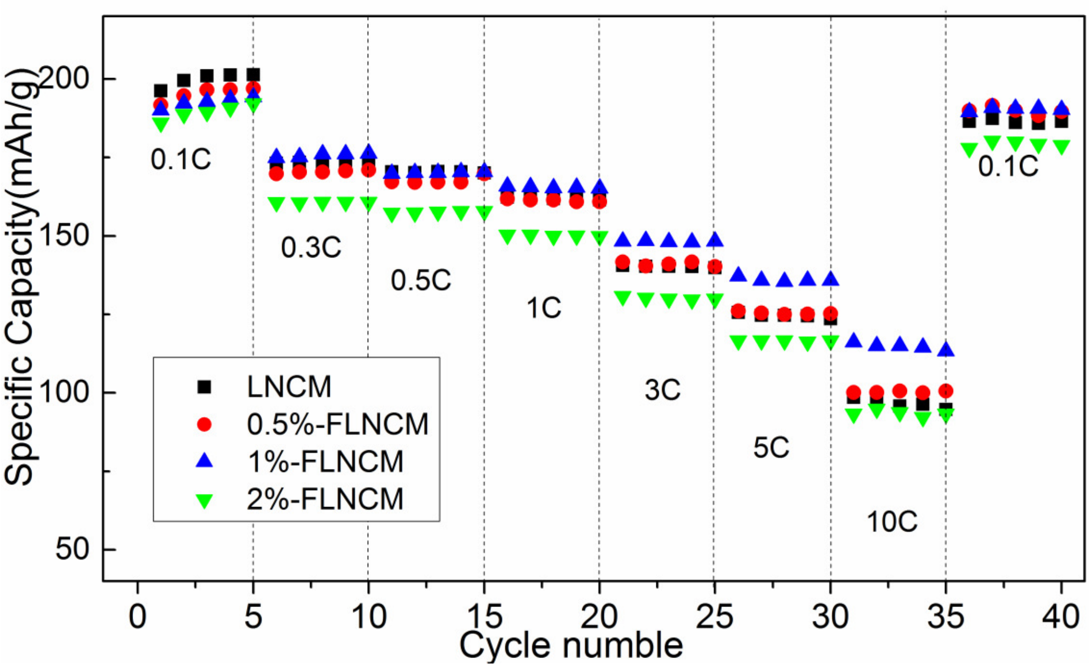

Figure S6. Rate capability of LNCM, 0.5%F-LNCM, 1%F-LNCM and 2%F-LNCM cathodes.

[1] W. Ludwig, P. Cloetens, J. Hirtwig, J. Baruchel, B. Hamelin, P. Bastie, J. Appl. Crystallogr. **2001**, 34, 602.
